# Polysilazane-Derived Layer for Enhancing Durability of Yttrium Oxyhydride Photochromic Coatings

**DOI:** 10.1021/acsomega.5c10912

**Published:** 2026-01-31

**Authors:** Vinoth Kumar Kasi, Elizaveta Shmagina, Sergei Bereznev, Ørnulf Nordseth, Jeyanthinath Mayandi, Smagul Karazhanov

**Affiliations:** † Department for Solar Energy Materials and Technologies, 11312 Institute for Energy Technology, P.O. Box 40, 2027 Kjeller, Norway; ‡ Department of Physics, 29944 Madurai Kamaraj University, Palkalai Nagar 1, 625 021 Madurai, India; § Virumaa College, School of Engineering, Tallinn University of Technology, Järveküla Tee 75, Kohtla-Järve 30322, Ida-Viru maakond, Estonia; ∥ Department of Materials and Environmental Technology, Tallinn University of Technology, Ehitajate Tee 5, 19086 Tallinn, Estonia; ⊥ Department of Chemistry, 29944 Madurai Kamaraj University, Palkalai Nagar 1, 625 021 Madurai, India; # Thin Film Laboratories, Institute of Solid State Physics, University of Latvia, Kengaraga Street 8, LV-1063 Riga, Latvia

## Abstract

Yttrium oxyhydride (YHO) exhibits photochromic properties under ambient conditions, making it a promising material for smart window applications. However, operational challenges including lattice contraction upon illumination, expansion during bleaching, and limited thermal stability introduce mechanical stress and susceptibility to degradation from environmental gases, compromising long-term performance. This study investigates perhydropolysilazane (PHPS)-derived SiO_x_N_y_ coatings as a solution for environmental protection of PHPS. A silicon–nitrogen–hydrogen preceramic polymer was deposited via spin coating and UV-cured on glass substrates, followed by reactive magnetron sputtering of YHO thin films. Three configurations glass/SiO_x_N_y_/YHO, glass/SiO_x_N_y_/YHO/SiO_x_N_y_, and glass/YHO/SiO_x_N_y_ were evaluated under solar simulator illumination, with optical performance characterized by UV–vis spectrophotometry. The results reveal that SiO_x_N_y_ coatings effectively shield YHO from environmental degradation while preserving its photochromic response. Minimal performance loss across coated architectures underscores the potential of PHPS-derived layers as multifunctional barriers, advancing the durability and applicability of YHO-based smart coatings.

## Graphical abstract

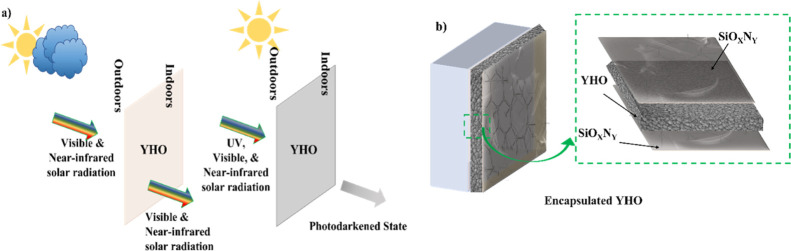

## 1 Introduction

The demand for energy-efficient and adaptive building technologies has grown significantly in recent years, driven by the need for sustainable solutions that enhance comfort and reduce energy consumption.^1^ Photochromic materials, which dynamically modulate light transmission in response to external stimuli, offer a promising avenue for smart window applications.^2–4^ Among them, yttrium oxyhydride YH_3–2x_O_x_ (YHO) has emerged as an attractive candidate due to its ability to transition from a transparent to a darkened state under ambient sunlight exposure. It is an inorganic photochromic material reported by Mongstad et al. in 2011 ^5^ and is extensively studied for a decade (see, e.g., refs 2, 3 , and^5–7^ ). Despite its potential, YHO faces critical challenges that hinder its practical implementation.

One of the key challenges is degradation arising from the intrinsic properties of the material. Prolonged illumination of YHO by an UV lamp and solar simulator can lead to the formation of hydride and oxide anion vacancies.^8–10^ Although these changes are partially reversible once the illumination ceases, they tend to slow down the bleaching process and significantly affect the photochromic performance, including contrast and bleaching kinetics. Additionally, extended light exposure may cause a gradual decrease in the transmittance of the photodarkened state.

The surrounding environment has been shown to play a crucial role in the photochromic behavior of YHO, although the current findings remain somewhat contradictory. YHO films exhibit significantly slower bleaching in a nitrogen atmosphere inside a glovebox,^5^ in vacuum conditions^11^ at room and low temperatures or when their front surface is sealed with a glass sheet. In contrast, rapid bleaching is observed in open air. Interestingly, YHO encapsulated with WO_3_ demonstrates reversible photochromism with enhanced performance, such as faster coloration and broader switching modulation compared to single-layer YHO,^12^ although the bleaching process remains relatively slow.^13^ Notably, this bilayer system also exhibits thermochromic behavior.^13^ Similarly, a YO_x_H_y_/VO_2_ bilayer deposited on quartz glass has shown both improved photochromic response under ambient conditions and thermochromism with a lowered transition temperature.^14^

Additionally, interactions with environmental gases also contribute to gradual degradation, limiting the long-term stability of YHO coatings.^6,15^ Although the YHO coating is covered with a ∼2 to 10 nm thin natural oxygen-rich layer at the surface,^16,17^ it is not protected well from the environment and the material can be subjected to both internal and external mechanisms of degradation. Furthermore, the effect of the oxygen-rich layer is not clear. Shielding from the negative influence of the environment^6,18^ is an important challenge. Inorganic thin films have been used for protecting the YHO coatings from the environment. The results obtained by different groups differ from each other. Encapsulation by a few tens of nanometer thick Si_3_N_4_ and Al_2_O_3_ deposited by magnetron sputtering did not change^7^ the photodarkening behavior of the films. Furthermore, the bleaching process for encapsulated and nonencapsulated YHO also showed similar relaxation time constants.^7^ Although protection of NdH_3–2x_O_x_ and GdH_3–2x_O_x_ by Al_2_O_3_ deposited by atomic layer deposition (ALD)^19^ has increased the lifetime of the films from one day to several months, on the other hand, the bleaching process in encapsulated coating became slow, and it was dramatically slowed down upon annealing. This finding is generally consistent with those reported in refs 20 and 21 , where YHO films encapsulated with approximately 30 nm of Al_2_O_3_ or TiO_2_ via ALD^20^ and around 40 nm of TiO_2_ deposited by ultrasonic spray pyrolysis^21^ exhibited a significantly extended bleaching time compared to nonencapsulated YHO films.

Addressing the challenges discussed above is crucial for advancing commercialization and widespread adoption of the photochromic YHO coating. The analysis presented above shows the importance of finding the best encapsulating material. Liquid preceramic polymer precursors, such as perhydropolysilazane (PHPS), hold great potential for safeguarding YHO. Being an inorganic material, PHPS is characterized by a silicon–nitrogen backbone, capable of forming dense, ceramic-like coatings upon exposure to air, moisture, or UV radiation.^22^ PHPS can easily be employed to produce high-quality thin vitreous silicon oxynitride (SiO_x_N_y_) layers^23–25^ by replacing initial nitrogen and hydrogen with oxygen via hydrolysis and condensation. SiO_x_N_y_ is a material known for its exceptional thermal stability, chemical resistance, and barrier properties. These characteristics make PHPS-derived coatings highly durable and suitable for demanding applications in electronics, aerospace, energy systems, and architectural glass.^26,27^ In addition to being transparent to sunlight (95%),^28^ PHPS is capable to bind covalently to the polar groups on natural or treated surfaces^29^ and it offers an excellent adhesion to many substrates, including metal, glass, plastics, ceramics, etc.^30,31^ The PHPS-derived films can be deposited by low cost and low-temperature fabrication methods^32^ forming smooth and uniform coatings on these substrates. Also, PHPS has found applications in transparent gas permeation barrier materials^33,34^ and water vapor barrier layers.^35^ Because of these impressive barriers and protective properties, PHPS is attractive for the YHO coatings as an encapsulant material.

This study investigates the application of a PHPS-derived SiO_x_N_y_ coating to mitigate the environmental resilience of YHO films. The impact of this encapsulation strategy on photochromic performance and optical properties is examined through prolonged illumination, UV–vis spectrophotometry. Results show that while SiO_x_N_y_ encapsulation slightly reduces the photochromic response of YHO, it markedly enhances the coating’s stability under extended exposure to sunlight.

## 2 Methods

Samples were prepared on microscope glass slides used as substrates. For SiO_x_N_y_ thin layer formation, a commercially available 20% solution of PHPS in dibutyl ether (NN-120-20, durXtreme GmbH, Germany) was used. The PHPS films were deposited by spin-coating using a Polos SPIN 150i programmable spin-coater (S.P.S. Ltd., The Netherlands) at 2000 rpm for 1 min. Before the deposition of the PHPS-derived binder layer, the glass substrates were washed in an ultrasonic bath for 5 min in isopropanol and then in a 20% Decon 90 solution. Next, the glass substrates were washed in Millipore water followed by drying under a flow of dry nitrogen of 99.995% purity. After the deposition of the PHPS film, the solvent residuals were evaporated on a hot plate at 40 °C for 2 min. Transformation (curing) of the PHPS to SiO_x_N_y_ was performed using UV-assisted technology as described in our previous work.^24^ The samples were irradiated with UV light with wavelengths of 185 and 254 nm simultaneously in air in a Novascan UV/Ozone cleaning system (Novascan Technologies Inc., USA). The distance between the samples and UV lamps was 15 mm. The curing time was 40 min.

The YHO films have been deposited following a two-step deposition process^36,37^ schematically represented in Figure 1. YH_2_ films were deposited by magnetron sputtering in argon and hydrogen in a Leybold Optics A550 V7 in-line sputtering system.^38^ In the second stage, the produced YH_2_ films were exposed to air, where they transformed into transparent and photochromic YHO. YHO films were deposited after SiO_x_N_y_ layer formation to obtain samples with structure glass/SiO_x_N_y_/YHO. For encapsulation, the PHPS layer was spin-coated on top of the YHO film and cured using the same UV-assisted method described above to obtain samples with structure glass/YHO/SiO_x_N_y_. In a similar process, the PHPS-coated glass first undergoes sputtering with a YHO layer and is then encapsulated with an additional PHPS coating. This combination provides samples with a glass/SiO_x_N_y_/YHO/SiO_x_N_y_ layered structure. The double encapsulation layer obtained by repeating the cycles of deposition/drying/curing of PHPS layers was also investigated in the samples with the name glass/YHO/SiO_x_N_y_/SiO_x_N_y_. So, in total, multilayer coatings with the following configurations were fabricated: glass/SiO_x_N_y_, glass/YHO, glass/SiO_x_N_y_/YHO, glass/YHO/SiO_x_N_y_, glass/SiO_x_N_y_/YHO/SiO_x_N_y_, and glass/YHO/SiO_x_N_y_/SiO_x_N_y_. The suitability of these encapsulating materials in real operating conditions was evaluated through transmittance and cyclic stability tests on the encapsulated photochromic film under UV illumination.

**Figure 1:**
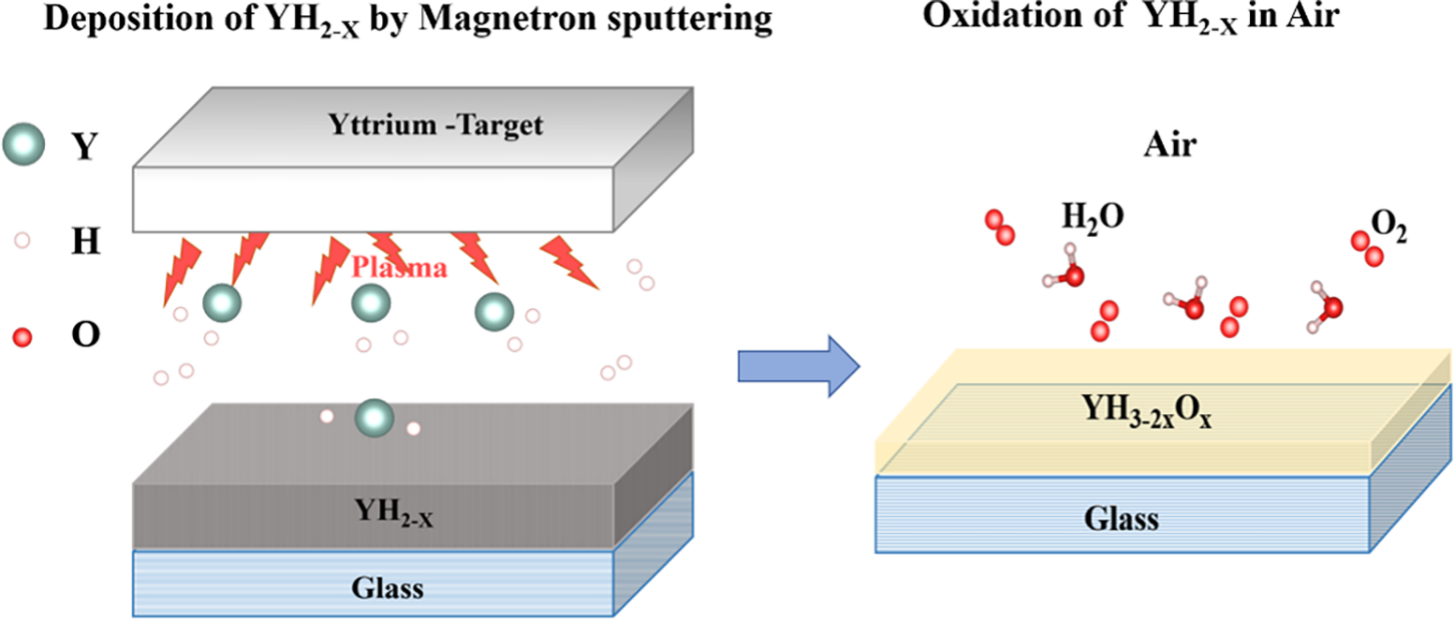
Schematic representation of the two-step deposition procedure for photochromic YHO thin films.

Thin film imaging was performed using an RTC-7 Inverted Tissue Culture Microscope (Radical Scientific Equipment’s Pvt. Ltd., Ambala, India) equipped with a long working distance condenser, plan achromatic phase contrast objectives (4×), and a trinocular camera port for photography. The optical properties, including transmittance (*T*) of the yttrium oxyhydride (YHO) films in both the clear and photodarkened states, were evaluated using an Ocean Optics QE6500 UV–vis spectrophotometer equipped with deuterium and tungsten halogen light sources.

Prior to the transmission measurements being set up, the instrument was calibrated in accordance with the standard calibration procedure relative to the air that was selected as the reference medium. The measurements were conducted in the wavelength range of 300–1000 nm. To investigate the photochromic properties, the samples were illuminated for 30 min using laser diode with a wavelength of 405 nm and power density of approximately 4.5 mW/cm^2^. For cyclical testing, the averaged optical transmission in the wavelength range of 650 to 850 nm was measured continuously for 30 min during the on and off states of the laser, repeated for 11 cycles.

Transmission spectra for the multilayer architectures glass/YHO/SiO_x_N_y_, glass/YHO/SiO_x_N_y_/SiO_x_N_y_, glass/SiO_x_N_y_/YHO, and glass/SiO_x_N_y_/YHO/SiO_x_N_y_ have been measured across the photon wavelength range of 350–950 nm for transparent, photodarkened after 10 h of illumination state, and bleached within 10 h state. Photodarkening of the coatings within 10 h has been implemented by using a broadband light source (EQ-99XFC LDLS), which also served as the probing beam due to its high intensity and strong UV component. To minimize unintended film darkening from this probing light, a long-pass colored glass filter (FGL850, Thorlabs) was employed to attenuate the beam. As a result, the measurement range was restricted to wavelengths between 600 and 1200 nm. Additionally, a 4.5 mW/cm^2^ violet laser (405 nm) was used as the excitation source to induce the photochromic effect.

## 3 Results and Discussion

Multilayer coatings with the following architectures were fabricated: glass/SiO_x_N_y_, glass/YHO, glass/SiO_x_N_y_/YHO, glass/YHO/SiO_x_N_y_, glass/SiO_x_N_y_/YHO/SiO_x_N_y_, and glass/YHO/SiO_x_N_y_/SiO_x_N_y_. Figure 2a–e displays microscope images of these multilayer coatings for qualitative comparison. The visual appearance of the coatings varies depending on the specific layer combinations of glass, SiO_x_N_y_, and YHO. This variability may be of practical interest, offering a potential method for tailoring the optical appearance of the coatings, an important consideration for meeting market demands.

**Figure 2:**
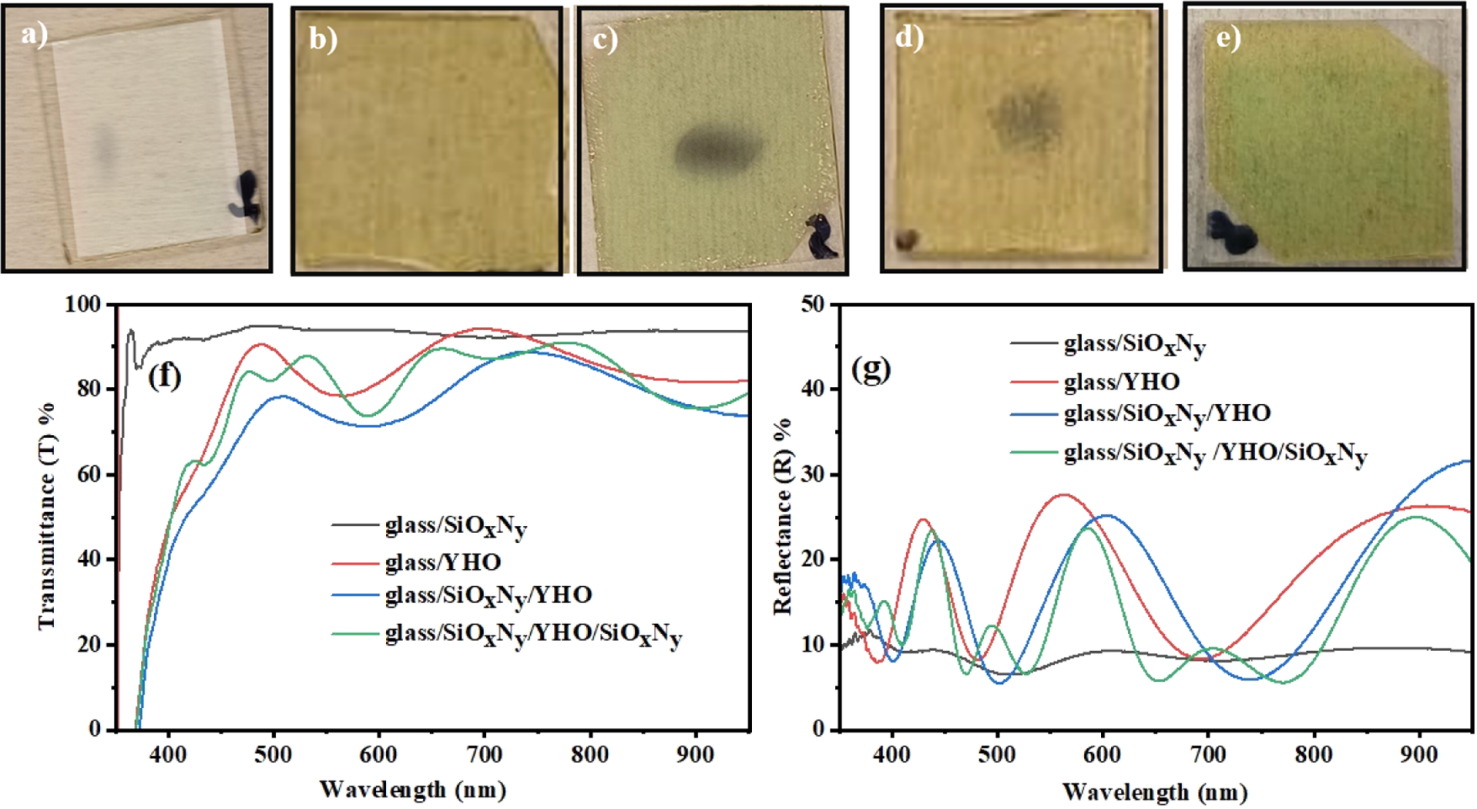
Optical microscope images of multilayer coatings: (a) glass/SiO_x_N_y_, (b) glass/YHO, (c) glass/YHO/SiO_x_N_y_, (d) glass/SiO_x_N_y_/YHO, and (e) glass/SiO_x_N_y_/YHO/SiO_x_N_y_. (f) Transmittance and (g) reflectance spectra for the multilayer coatings glass/SiO_x_N_y_, glass/YHO, glass/YHO/SiO_x_N_y_, and glass/SiO_x_N_y_/YHO/SiO_x_N_y_.

Across the photon wavelength range of 350–950 nm, the configurations demonstrate an average transmittance of >80% [Figure 2f] and reflectance around 15% [Figure 2g]. There is room for tuning of optical performance of the architectures by thickness of the SiO_x_N_y_ layers as well as the YHO. The coatings were subjected to cyclic illumination with UV light—30 min of exposure followed by 30 min in the dark—for a total of 11 cycles during the working day. All samples exhibited photochromic contrast exceeding 30%. Following the final illumination cycle, the films returned to their bleached (transparent) state within approximately 12 h, by the morning of the next day.

The photochromic performance of YHO coatings is highly vulnerable to environmental exposure when they are not encapsulated, which can lead to progressive degradation. Figure 3a illustrates the transmission spectra of YHO/glass coatings in both transparent and photodarkened states, comparing freshly deposited films with those stored in open air for four months. The as-prepared film exhibited an average transmittance of ∼84% in the transparent state and ∼20% in the photodarkened state. After four months of storage in the air, transmittance of the transparent state increased to ∼87% suggesting oxidation of the film over time. More strikingly, the transmittance of the photodarkened state after 30 min of illumination increased significantly to ∼67%, indicating a severe reduction in photochromic response. These results underscore the critical importance of encapsulating YHO coatings to maintain their optical performance and long-term stability.

**Figure 3:**
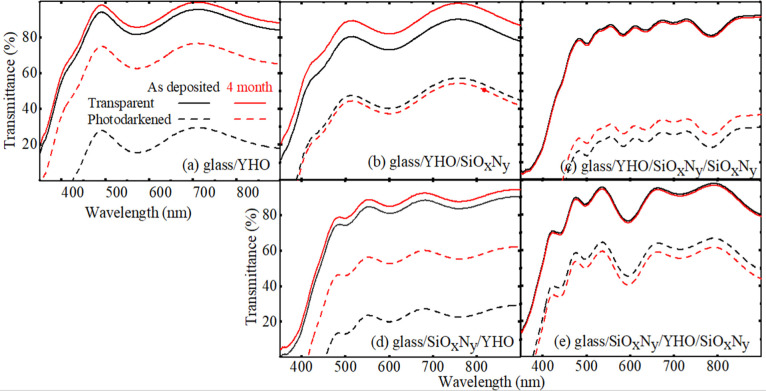
(a) Transmittance spectra for transparent (―) and photodarkened (---,--- (red)) states of as-deposited YHO/glass (―,---) as compared to that measured after 4 months (— (red),--- (red)). Transmittance spectra for (b) glass/YHO/SiO_x_N_y_, (c) glass/YHO/SiO_x_N_y_/SiO_x_N_y_, (d) glass/SiO_x_N_y_/YHO, and (e) glass/SiO_x_N_y_/YHO/SiO_x_N_y_ architectures for the transparent state (—) measured prior to UV illumination, the photodarkened states (···,··· (red)) after 10 h of illumination, and the bleached state (— (red)) after 10 h.

Figure 3b–e presents the transmission spectra for various multilayer architectures: glass/YHO/SiO_x_N_y_, glass/YHO/SiO_x_N_y_/SiO_x_N_y_, glass/SiO_x_N_y_/YHO, and glass/SiO_x_N_y_/YHO/SiO_x_N_y_, measured across the photon wavelength range of 350–950 nm for the transparent state and photodarkened state after 10 h of illumination for the deposited film as compared to that after 4 months of storage in a box under atmospheric air. The analysis reveals that the effectiveness of protection depends significantly on the thickness of the SiO_x_N_y_ encapsulant. The YHO coating shield by a single SiO_x_N_y_ layer of approximately ∼350 nm exhibited notable degradation [Figure 3b]. While the transmittance in the bleached state decreased only slightly compared to the transparent state, the photodarkened state showed a marked increase in transparency relative to the as-deposited sample, indicating a reduced optical contrast. This degradation is likely due to insufficient encapsulant thickness; pores and microdefects formed during the deposition of polysilazane may allow oxygen and moisture to infiltrate the YHO layer. To address this issue, an additional polysilazane layer was applied to enhance the protective performance. Indeed, the glass/YHO/SiO_x_N_y_/SiO_x_N_y_ architecture encapsulated with a double SiO_x_N_y_ layer (∼700 nm) exhibited minimal degradation [Figure 3c].

The architecture incorporating PHPS-derived layers as an encapsulant from the front and bottom sides [Figure 3e] exhibited noticeably less degradation of photochromic performance as compared to those employing perhydropolysilazane solely as an encapsulant from the front side. This enhanced optical behavior indicates a more stable and consistent modulation of light transmittance, preserving the contrast between states over time. The improved performance is likely a result of protecting the YHO layer. These findings underscore the importance of the encapsulation strategy from the front and bottom side in achieving durable and efficient photochromic coatings.

The application of PHPS-derived protecting layers in photochromic YHO (yttrium oxyhydride) coatings does not significantly affect the kinetics of color change or bleaching. Figure 4 presents the photodarkening and bleaching behavior of as-deposited films compared to those stored in air for four months. As shown in Figure 4a, the photodarkening time remains nearly unchanged after storage, indicating stable coloration kinetics. However, the bleaching process accelerates notably after four months, suggesting degradation. Specifically, the transmittance in the photodarkened state decreases from ∼30% in the as-deposited film to ∼70% after storage, pointing to a substantial loss of photochromic contrast in unencapsulated YHO coatings. In contrast, films encapsulated from the front side with SiO_x_N_y_ exhibit consistent transmittance levels and coloration/bleaching kinetics before and after aging, as seen in Figure 4c,b. Notably, Figure 4d demonstrates minimal changes in both photodarkening and bleaching rates for YHO films treated with SiO_x_N_y_ from both the front and bottom sides. Unlike many inorganic photochromic coatings, where encapsulation with inorganic compounds from the front side often leads to prolonged bleaching times,^13,14,19–21^ SiO_x_N_y_ encapsulation has a negligible impact on bleaching duration. Furthermore, the protective performance of SiO_x_N_y_ is dependent on its thickness. As illustrated in transmittance spectra [Figure 3c], a thicker SiO_x_N_y_ layer offers superior protection against degradation compared to a thinner one, as evidenced by the comparison between Figures 3b and 3c. These findings highlight the effectiveness of SiO_x_N_y_ encapsulation in preserving the photochromic functionality of YHO coatings over time, see Figure 4d.

**Figure 4:**
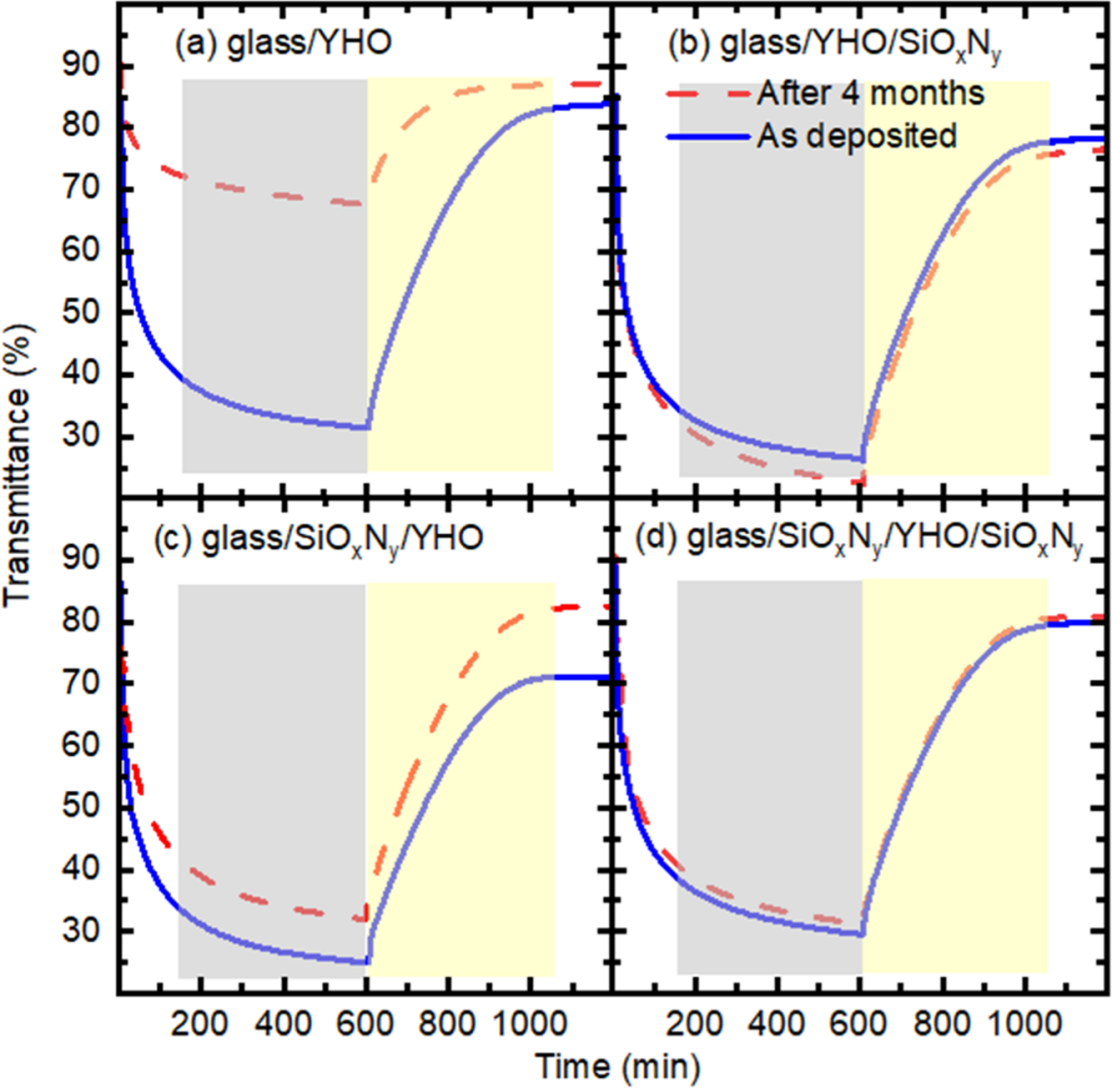
Kinetics of color change and bleaching for (— (blue)) as-deposited films and (--- (red)) after 4 months for the architectures: (a) glass/YHO, (b) glass/SiO_x_N_y_/YHO, (c) glass/YHO/SiO_x_N_y_, and (d) glass/SiO_x_N_y_/YHO/SiO_x_N_y_.

Polysilazanes, known for their excellent film-forming and barrier properties,^29,38^ may enhance the environmental stability of YHO coatings and serve as effective barriers against moisture and oxygen—two factors known to accelerate degradation or disrupt the hydrogen exchange central to YHO’s photochromic behavior. Additionally, their ability to form dense ceramic materials upon curing could affect hydrogen diffusion dynamics, an essential factor in the photochromic response of YHO materials. A recent study has shown the potential of PHPS-derived films as a hydrogen permeation barrier.^26^ By tailoring the interface and limiting hydrogen loss or ingress, PHPS-derived layers might stabilize the bleaching process, leading to a more consistent optical performance under varying illumination conditions.

## 4 Conclusion

In summary, multilayer configurations YHO/SiO_x_N_y_/glass, SiO_x_N_y_/YHO/glass, and SiO_x_N_y_/YHO/SiO_x_N_y_/glass were fabricated and evaluated, revealing that the integration of SiO_x_N_y_ shields YHO from degradation. In the transparent state, the configurations demonstrated high average transmittance (>80%) and moderate reflectance (∼15%) across the visible spectrum. Under cyclic UV illumination, they exhibited robust photochromic behavior with contrast exceeding 30% and reliably returned to their transparent state during the night with minimal loss in photochromic functionality. This study demonstrates the effectiveness of PHPS-derived SiO_x_N_y_ layers as protecting layers in multilayer photochromic YHO coatings. These findings underscore the critical role of interface engineering and encapsulation strategy in advancing YHO-based smart coatings. By enabling low-temperature processing and improving environmental resilience, this approach offers a viable pathway for advancing YHO materials toward scalable and long-term applications in energy-efficient building technologies and adaptive optical devices. In defining the relevance of these multilayer configurations for practical applications, it is important to note that the optimal stack architecture depends on the performance metrics required for specific use scenarios. For building-integrated applications, desirable criteria typically include high visible transmittance in the bleached state, sufficient solar modulation capability, fast and reversible switching, long-term environmental stability, and mechanical robustness under repeated cycling. Greenhouse glazing, in contrast, may prioritize spectral selectivity and minimal reflectance to maintain photosynthetically active radiation while still providing dynamic shading capability. Within the scope of the present work—limited to optical performance and qualitative cycling behavior, the SiO_x_N_y_/YHO/SiO_x_N_y_/glass configuration appears the most promising, as it provides environmental protection while maintaining high transmittance and strong photochromic contrast. However, a definitive determination of the optimal configuration will require future studies incorporating standardized mechanical testing, durability assessments, adhesion and scratch resistance measurements, and long-term cycling under realistic operating conditions. Such evaluations will enable a comprehensive connection between application-specific performance metrics and functional coating architecture.

## Data Availability

Due to the large amount of data related to the calculation results in this paper and the limitation of the data-sharing conditions of our institution, it is not possible to upload all the data to the public network. We can share the relevant calculations and know how upon contacting the corresponding author of the paper.
